# Best practice approach to the risk management of Legionella infection in health care facilities

**DOI:** 10.1186/1753-6561-5-S6-P305

**Published:** 2011-06-29

**Authors:** B Casini, A Buzzigoli, P Valentini, A Vecchione, F Torracca, A Baggiani, G Privitera

**Affiliations:** 1 Dep. Esperimental Pathology, MBIE, University of Pisa, Pisa, Italy; 2 U.O. Igiene ed Epidemiologia, Azienda Ospedaliero-Universitaria Pisana, Pisa, Italy

## Introduction / objectives

Aim of this study is to summarize and re-evaluate our eight year experience in the application of a water safety plan (WSP) in a 1500-bed teaching hospital.

## Methods

WSP is based on a strategy integrating surveillance, maintenance, continuous chlorine-based disinfection and end-point filtration in critical areas. The strategy includes also molecular typing of the isolates and virulence genes expression analysis to detect the occurrence of adaptative changes in strains colonizing the water system.Recently, monochloramine treatment was applied in selected areas.

## Results

Before the disinfection-filtration strategy, Legionella was isolated in 67% of samples (54/81), 79% of these samples exceeding 10^3^ CFU/L. After eight-years of integrated strategy, Legionella was still present but the positive supply points were reduced to 22% (54/241) in the last year, and the samples exceeding 10^3^ CFU/L were cut down to 18%. All isolates were identified as *L. pneumophila* sg1, two predominant and persisting clones, one of which showing increased chlorine tolerance. Long-time exposure to chlorine enhanced the ability to express more promptly some virulence genes involved in intracellular protozoa infection. The application of end-point filtration in high-risk areas is therefore required until a new effective disinfectant is introduced. Following the substitutions of chlorine dioxide by monochloramine, eradication of planktonic Legionella was observed although long-time effects have to be evaluated.

## Conclusion

Standard environmental surveillance methods may not be sufficient to determine the most effective disinfection method and should be accompanied by evaluation of the susceptibility to sanitising agents.

## Disclosure of interest

None declared.

